# Study on Tribological Characteristics of Ultra-High Molecular Weight Polyethylene under Unsaturated Lubrication of Water and Brine

**DOI:** 10.3390/polym14194138

**Published:** 2022-10-03

**Authors:** Wenhao Li, Zhenhua Wang, Ningning Liu, Jinzhu Zhang

**Affiliations:** 1 College of Water Conservancy & Architectural Engineering, Shihezi University, Shihezi 832000, China; 2 Key Laboratory of Modern Water-Saving Irrigation of Xinjiang Production & Construction Corps, Shihezi University, Shihezi 832000, China

**Keywords:** ultra-high molecular weight polyethylene, tribological characteristic, unsaturated lubrication

## Abstract

The tribological characteristics of ultra-high molecular weight polyethylene (UHMWPE) under unsaturated lubrication of water and brine were studied. The friction coefficients and wear rates of UHMWPE at different applied loads and sliding speeds were recorded by field tests, and the effects of load and speed on the friction properties of UHMWPE were analyzed. The results showed that under certain liquid drop (about 150–170 mL/h) lubrication, the tribological behaviors of UHMWPE were better than those of dry sliding, and the friction coefficient and wear rate of UHMWPE were reduced by more than 39% and 10% respectively. The lubrication form of UHMWPE gradually transited from saturated lubrication to unsaturated lubrication with the increase in applied load or sliding speed. The evaporation of water caused by frictional heat affected the water content between the surface of UHMWPE and the counterface, which was the main reason for the change in the lubrication form. In the current work, the critical values for the change of lubrication mode were 70 N and 700 r/min for load and speed, respectively, beyond which UHMWPE was in unsaturated lubrication. Under brine-unsaturated lubrication, the anti-friction property of UHMWPE was better than that in water-unsaturated lubrication at high speed because the precipitated salt granules played a ball effect, which was opposite to that under saturated lubrication. The study of the wear resistance with surface profiler showed that the wear rate of UHMWPE under water-unsaturated lubrication was 9% lower than that under brine-unsaturated lubrication at 110 N load. While the wear resistance of UHMWPE under brine-unsaturated lubrication was better than that in water-unsaturated lubrication at high speed, the wear rate of UHMWPE under brine-unsaturated lubrication was 10% lower than that under water-unsaturated lubrication at 1100 r/min speed.

## 1. Introduction

Ultra-high molecular weight polyethylene (UHMWPE) is a high-performance polymer matrix composite material with high toughness, high strength, high modulus and low density [1]. It is considered as one of the most ideal reinforcement materials and has played a vital role in many fields due to its excellent comprehensive properties such as chemical stability, bio-compatibility and abrasion resistance [2,3,4]. Specifically, UHMWPE has a wide range of applications in the fields of military industry, mechanical engineering and medicine due to its excellent friction response characteristics [5,6,7,8]. In a liquid environment, due to the certain water absorption of UHMWPE, boundary lubrication regimes are formed on its surface, which significantly reduce the friction coefficient of UHMWPE [9], and many scholars have paid attention to the tribological properties of UHMWPE under different liquid lubrication conditions. Cooper et al. [10] studied the effect of different tribological conditions on the wear processes under deionized water lubrication condition using two tri-pin-on-disc apparatuses. Xiong and Ge [11] investigated the tribological properties of UHMWPE sliding against ceramic under lubrication of distilled water, saline and fresh plasma. Chang et al. [12] evaluated the effect of accelerated aging on the mechanical and tribological properties of UHMWPE.

Other meaningful research has been conducted on the friction characteristics of UHMWPE under different lubrication, and the friction mechanism has been fully investigated [13,14,15,16]. Most of the existing studies were carried out under saturated lubrication in a liquid bath, and less attention is being paid to tribological properties of UHMWPE under unsaturated lubrication. Wang et al. [9] studied the friction and wear characteristics of UHMWPE composites under water lubrication, which was applied by dropping distilled water onto the sliding surface and was treated as saturated lubrication. However, unlike a liquid bath, the evaporation of water due to frictional heat can cause changes in the water content between the surface of UHMWPE and the counterface at a certain drop lubrication rate, which will affect the friction characteristics of UHMWPE.

The friction heat is directly affected by applied load and sliding speed [17]. Therefore, the main purpose of this experiment is to study the tribological characteristics of UHMWPE at different loads and speeds under unsaturated lubrication. The friction and wear properties of UHMWPE sliding against steel ball under unsaturated lubrication of water and brine were investigated. For comparison, wear tests were performed under dry condition.

## 2. Materials and Methods

### 2.1. Materials

The UHMWPE friction samples were manufactured by the Anyang City Ultra-high Industrial Technology Co., Ltd., Anyang, China. The samples were 15 mm diameter cylinders with a thickness of 5 mm. The basic technical performance is shown in Table 1. Friction antithesis was 45# steel ball with a diameter of 5 mm. After hardening and tempering, its hardness was HRC65 and surface roughness was Ra0.11 μm. The brine lubricating liquid used in the test was prepared from the soil leaching solution of the test field of Shihezi Paotai Town, Shihezi, China. The salinity of the solution is 7 g/L, and the main ionic component contents are shown in Table 2. Pure water was obtained with an ultrapure water machine (Shanghai Leading Water Treatment Equipment Co., Ltd., Shanghai, China, electric resistance rate of 18.25 MΩ·cm at 25 °C).

### 2.2. Experimental Device

**Figure 1:**
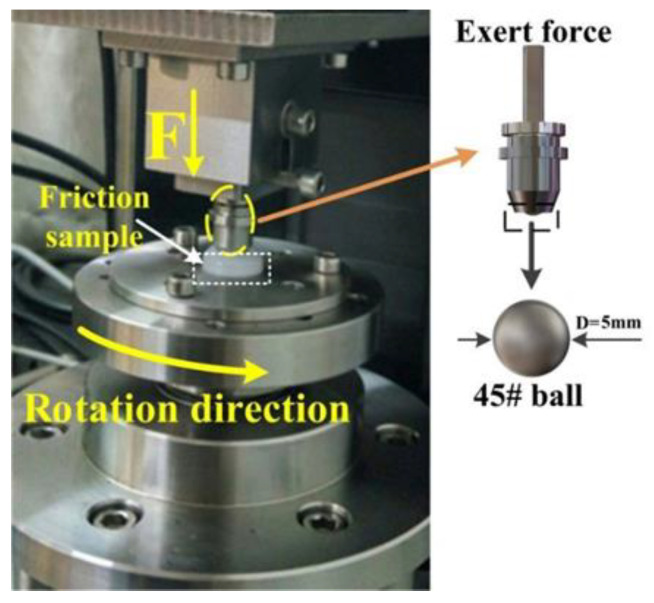
Schematics of the test configuration.

The experiments were conducted at room temperature 20 °C and 35% relative humidity. Before the test, in order to reduce the influence of refined grain abrasion, 800# metallographic sandpaper was used to polish the surface of the steel ball and the samples. The test sample was fixed on the bottom chassis of the CFT-I multifunctional material surface comprehensive performance tester (Lanzhou Zhongke Kaihua Technology Development Co, Ltd., Lanzhou, China), which would record 90 data per minute and draw the friction coefficient curve on the LCD screen, and the steel ball was mounted on the tester arm, as shown in Figure 1. The UHMWPE/steel ball friction wear test began when the sample remained stationary and the friction pairs slid against each other, and the rotating diameter was 5 mm. Unsaturated lubrication was applied by dropping water or brine onto the sliding surface at a flow rate of 55–60 drops per minute (about 150–170 mL/h).

AUW220D electronic balance (Shimadzu Company, Shimane, Japan) was used to measure the weight of the sample. After the friction test of UHMWPE under the liquid lubrication condition, the residual lubricant on the surface of UHMWPE was absorbed with fiber absorbent cotton, and after natural drying, the carbon film was sprayed on the surface of UHMWPE by EMS150RE vacuum coater (Electron Microscopy Sciences Company, Hatfield, PA, USA) for observation in scanning electron microscope (SEM), and SIGMA 300 scanning electron microscope (Carl Zeiss, Oberkochen, Germany) was used to observe the worn surface morphology of UHMWPE samples. GENESIS 2000 X-ray spectrometer (EDAX Co., Ltd., Pleasanton, CA, USA) was used to analyze the distribution of elements on the worn surface of samples. ST400 surface profiler (NANOVEA, Irvine, CA, USA) was used to analyze the micro-morphology of the worn surface to determine the wear of UHMWPE samples under different experimental conditions. The hand-held infrared temperature gun was used to record the temperature of the UHMWPE surface every 30 s during the test.

## 3. Results and Discussion

**Figure 2:**
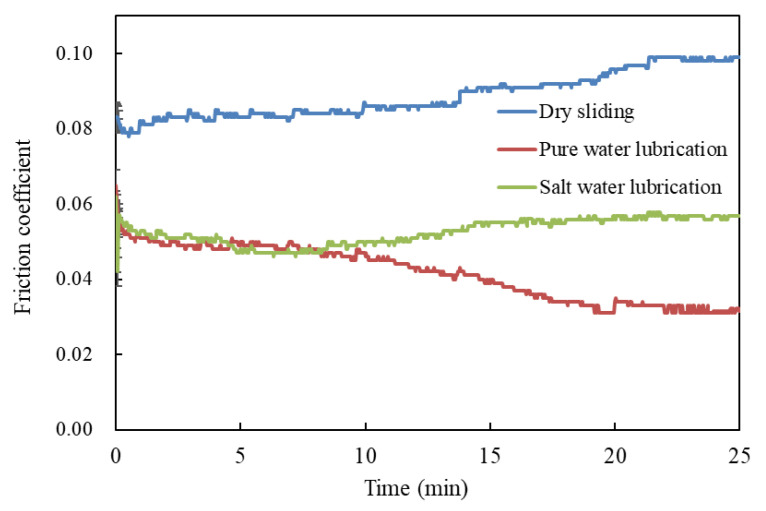
Variation of friction coefficient along with time under different lubrications.

Figure 2 shows the relation between the friction coefficient and time for the different lubrication conditions at 500 r/min speed and 70 N applied load. The friction coefficient changed with time at the beginning of the test but then reached a stable value. The steady-state friction coefficients showed a maximum value (approximately 0.10) for dry sliding and minimum (about 0.032) for water lubrication. The water absorption resulted in the swelling of UHMEPE and decreased the shear strength of UHMWPE, thus forming boundary lubrication regimes that reduced the friction coefficient [9, 11], and the water absorption in brine was lower than that in water; thus, the friction coefficient of UHMWPE was higher than that in the water lubrication condition [11].

After sliding for about 20 min, under lubrication of water and brine, the friction coefficient reached steady state as the water absorption rate of UHMWPE surface entered steady state, and the friction coefficient of the dry condition reached steady state after sliding for about 22 min due to the stability of the transfer film, which strongly depended on time and temperature [17]. Under other pressures and speeds, the friction coefficient could reach a stable state within 25 min. Therefore, the friction coefficient values in this paper were recorded after 25 min of the test.

### 3.1. Effect of Applied Load on Friction Coefficient under Unsaturated Lubrication of Water and Brine

**Figure 3:**
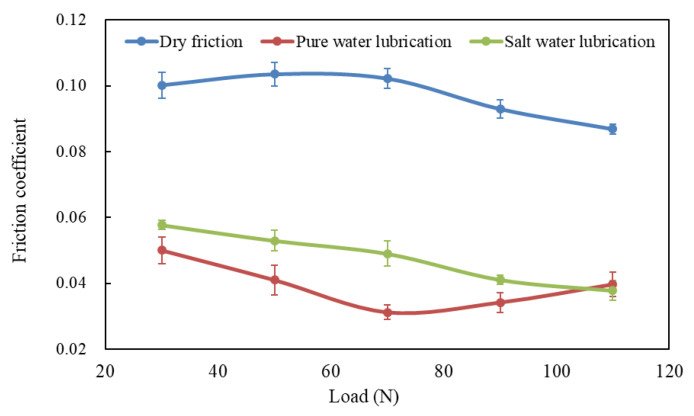
Variation of friction coefficients of UHMWPE with load at 500 r/min speed under different friction conditions.

Figure 3 shows the variation of friction coefficients of UHMWPE at 500 r/min speed and 30, 50, 70, 90, 110 N applied loads in dry, water-unsaturated lubrication and brine-unsaturated lubrication conditions. For UHMWPE sliding against steel ball under dry conditions, the friction coefficient decreased with the increase in applied load. The trend has been described using the equation in [18]:

*μ* = *kL*^(*n* − 1)^, (1)

where *μ* is the friction coefficient, *L* the load, *k* a constant and *n* a constant. According to the formula, the friction coefficient decreased with increasing load [19].

**Figure 4:**
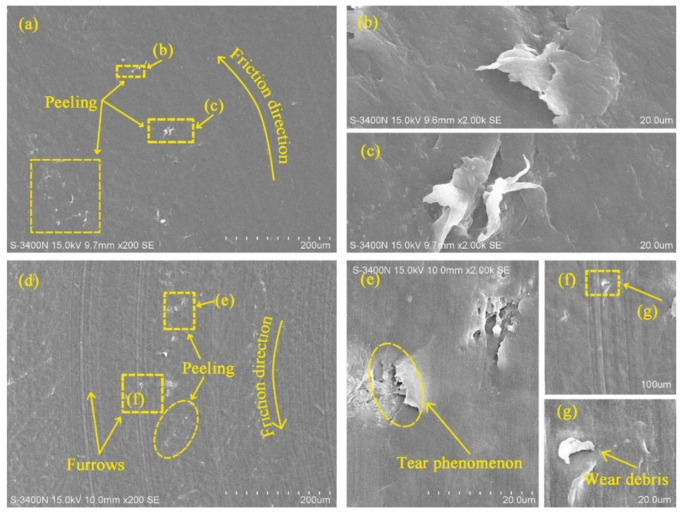
SEM micrographs of worn surfaces of UHMWPE under water-unsaturated lubrication (**a**) 50 N load and (**d**) 110 N load; (**b**,**c**) and (**e**–**g**) are the images taken at the marked positions in the micrographs.

**Figure 5:**
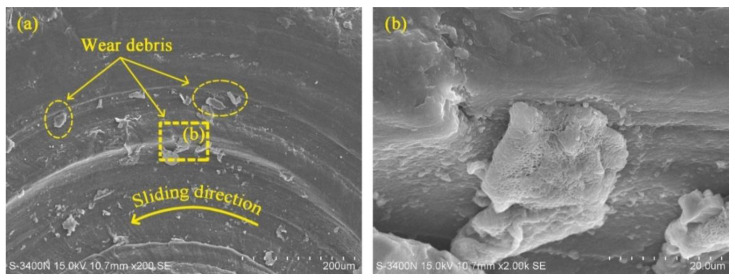
SEM micrographs of worn surface of UHMWPE under dry condition at 110 N applied load. (**b**) is the image taken at the marked position in (**a**).

The variation of friction coefficient with load under water-saturated lubrication also followed the equation in [9]. In other words, the friction coefficient of UHMWPE decreased with the increasing load under water-saturated lubrication. However, there are different phenomena in Figure 3 that the friction coefficient of UHMWPE first decreases and then increases with the increasing load under water-unsaturated lubrication. The worn surfaces of UHMWPE were examined using SEM. Figure 4 shows the SEM micrographs of worn surfaces of UHMWPE under water-unsaturated lubrication at 50 and 110 N loads. It can be seen that the surface of the sample is smooth, and there is only peeling on the surface at 50 N load, as shown in Figure 4a–c. However, peeling, furrows, tear fracture and wear debris are found on the surface at 110 N load (Figure 4d–g), and the profile of the debris in Figure 4g is similar to the profile of the debris found on the sample surface under dry condition, as shown in Figure 5.

Therefore, under certain water drop lubrications, UHMWPE gradually transited from saturated lubrication to unsaturated lubrication with the increase in load, and the critical load was 70 N in this paper, beyond which UHMWPE samples were in unsaturated lubrication. The main reason for the phenomenon was the evaporation of water caused by frictional heat. When the load was less than the critical value, the water evaporation rate due to frictional heat was much smaller than the water drop rate, and the UHMWPE samples were in a saturated lubrication state. When the load exceeded the critical value, the temperature rise effect caused by the load increase could not be ignored (as shown in Table 3), the water evaporation rate was higher, and the water content between the surfaces of UHMWPE and counterface decreased with the increase in load, which in turn caused the friction coefficient to increase. On the whole, water-unsaturated lubrication significantly improved the anti-friction performance of UHMWPE, and the friction coefficient of UHMWPE decreased by more than 50% compared with that of dry sliding.

It can be seen from Table 3 that the surface temperature of UHMWPE under brine lubrication is basically the same as that under water lubrication. Due to the same dripping rate as that of water lubrication, it was considered that the UHMWPE also transitioned from saturated lubrication to unsaturated lubrication with the increase in load under brine lubrication, and the critical load was also 70 N. In the load range of 50–110 N, the friction coefficient of brine-unsaturated lubrication was more than 42% lower than that of dry sliding.

**Figure 6:**
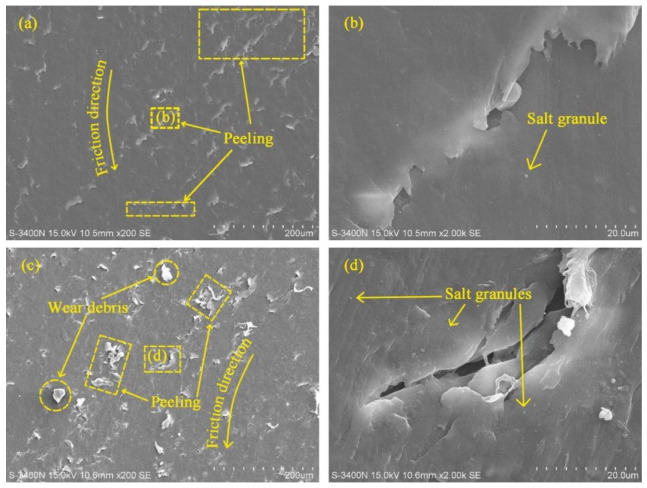
SEM micrographs of worn surfaces of UHMWPE under brine-unsaturated lubrication conditions (**a**) 50 N load and (**c**) 110 N load; (**b**,**d**) are images taken at the marked positions in the micrographs.

**Figure 7:**
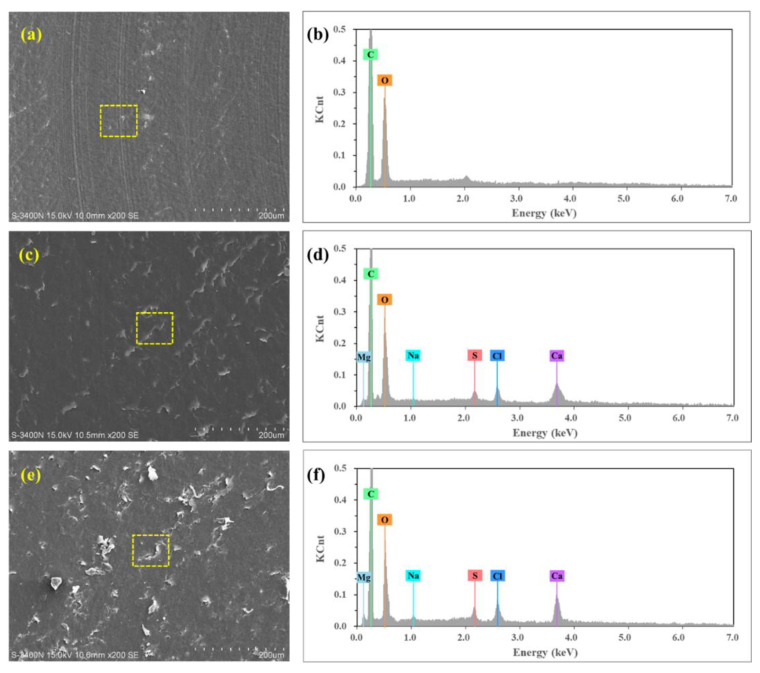
EDS analysis of worn surface of UHMWPE (**a**) water-unsaturated lubrication with 110 N load, (**c**) brine-unsaturated lubrication with 50 N load, and (**e**) brine-unsaturated lubrication with 110 N load; (**b**,**d**,**f**) are EDS spectra of the sample surface labeled in (**a**,**c**,**e**), respectively.

Figure 6 shows SEM micrographs of the worn surface of UHMWPE under brine-unsaturated lubrication at 50 and 110 N loads. In the sample at 50 N load (Figure 6a), very few granules were observed, and there were many peels on the surface of the sample. At 110 N load, there were more granules on the surface of the sample (Figure 6c). Elements Mg, Na, S, Cl and Ca were found in the area where granules appeared under brine-unsaturated lubrication, while these elements were absent under pure water-unsaturated lubrication, as shown in Figure 7, and the count of these elements increased with the increase in load. Therefore, it can be judged that the granules on the surface of UHMWPE are salt particles under brine-unsaturated lubrication.

This indicated that as load increased, the increasing frictional heat led to an increase in water evaporation rate. Therefore, precipitated salt granules increased with increasing load. The granules were inevitably pressed into the sample under the action of external force, resulting in micro scratches on the surface of the sample. Although this worsened the wear performance of UHMWPE (to be mentioned later), these granules still acted as rolling balls, reducing the friction coefficient. Therefore, for brine-unsaturated lubrication, the friction coefficient of UHMWPE monotonic decreased with increasing load, as shown in Figure 3.

### 3.2. Effect of Speed on Friction Coefficient under Unsaturated Lubrication of Water and Brine

**Figure 8:**
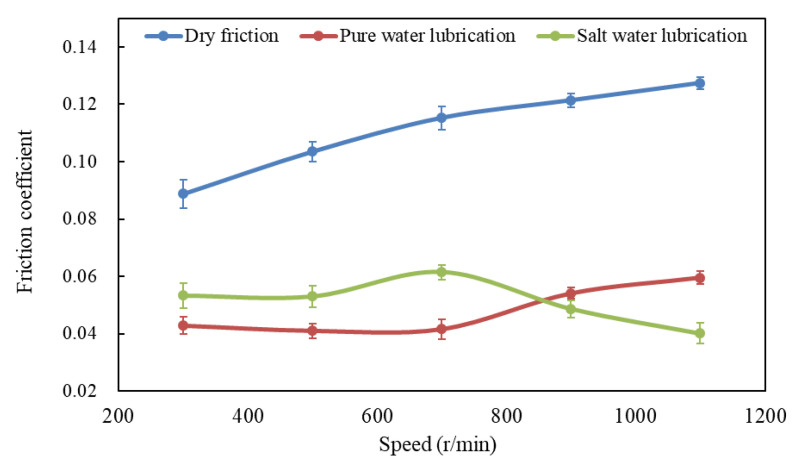
Variation of friction coefficient with speed under different friction conditions.

Figure 8 shows the variation of frication coefficient with speed under dry, water-unsaturated lubrication and brine-unsaturated lubrication conditions at 50 N load and 300, 500, 700, 900 and 1100 r/min speeds, and it can be seen that the friction coefficient under liquid lubrication is 39% less than that under dry friction in the speed range. For UHMWPE sliding against steel ball under dry conditions, the friction coefficient increased with increasing speed, as shown in Figure 7.

**Figure 9:**
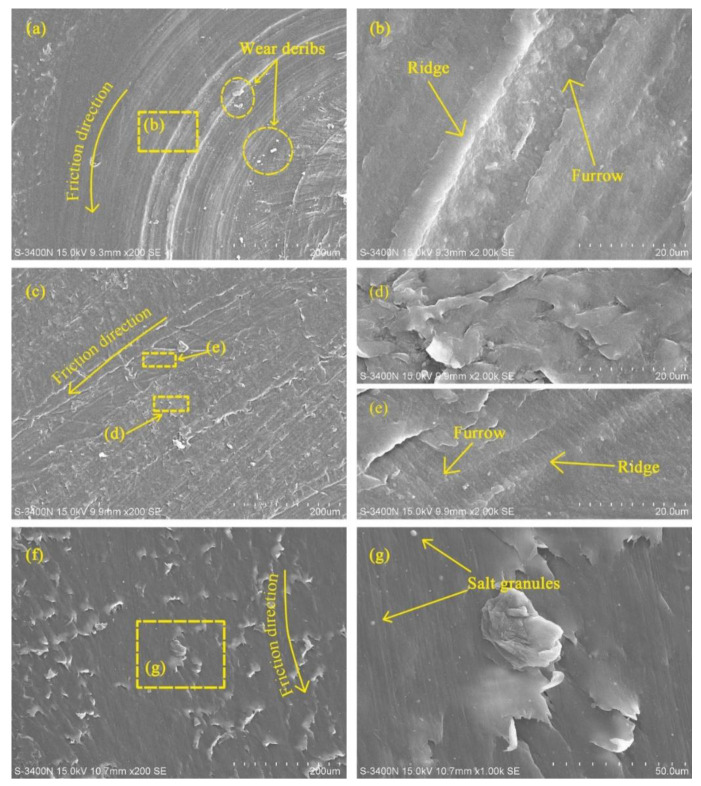
SEM micrographs of worn surfaces of UHMWPE at 1100 r/min speed (**a**) dry friction, (**c**) water-unsaturated lubrication and (**f**) brine-unsaturated lubrication; (**b**,**d**–**e**,**g**) are the images taken at the marked positions in the micrographs.

The variation of frication coefficient with speed under water-unsaturated lubrication was basically stable at first and then increased with the increasing speed, as shown in Figure 8. Compared with the SEM micrograph of UHMWPE at 50 N load and 500 r/min speed (Figure 4a), it can be seen that the surface of UHMWPE under water-unsaturated lubrication has not only peeling but also furrows and ridges at 50 N load and 1100 r/min speed, and similar furrows and ridges were found on the surface of UHMWPE under dry sliding at 50 N load and 1100 r/min speed (Figure 9a,c).

Similar to load, frictional heat played an important role in the influence of speed on the friction coefficient of UHMWPE under water-unsaturated lubrication. When the speed was low (<700 r/min in this experiment), the rate of water evaporation due to frictional heat was lower than the water drop rate. Therefore, the surface of UHMWPE was in a saturated lubrication state. At a higher speed, more heat was generated per unit time, and the surface temperature of UHMWPE increased rapidly (as shown in Table 4), leading to most of the dripped water evaporation. Only a small amount of water was absorbed by the surface of UHMWPE, which resulted in the sample surface being a state between water-saturated lubrication and dry sliding. As the speed increased, the water-unsaturated lubrication state approached the dry-slip state, the friction coefficient of UHMWPE increased, and furrows and ridges appeared on the UHMWPE surface due to thermal deformation and compression.

**Figure 10:**
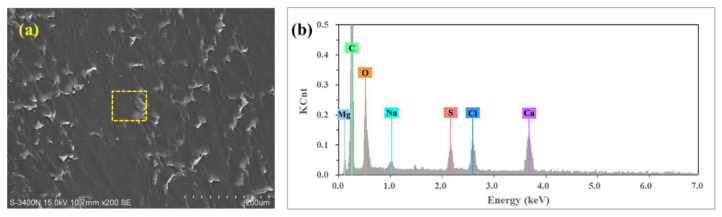
EDS analysis of worn surface of UHMWPE under brine-unsaturated lubrication with 1100 r/min speed, where (**b**) is EDS spectra of the sample surface labeled in (**a**).

Some salt granules are found on the surface of UHMWPE at 1100 r/min speed and 50 N load under brine-unsaturated lubrication, as shown in Figure 9g, and the variation of the friction coefficient illustrated that the effect of speed on the friction coefficient of UHMWPE under brine-unsaturated lubrication was affected by the combined effect of the evaporation rate of water and the precipitation rate of salt granules. When the speed was low, there were few salt granules precipitated, and the key factor affecting the frication coefficient was the water content between the surface of UHMWPE and the counterface. As the speed increased, the rate of water evaporation increased, and the lubricating effect of brine decreased, causing the friction coefficient to increase. When the speed exceeded the critical value (700 r/min in present work), a large number of salt granules were precipitated out. It can be seen from Figure 10 that the counts of elements Mg, Na, S, Cl and Ca on the sample surface at 1100 r/min speed are more than those at 110 N load, and the effect of the rolling balls on the friction coefficient was dominant. With the increase in speed, the friction coefficient under brine-unsaturated lubrication decreased, and the anti-friction properties superior to water-unsaturated lubrication were obtained in the high-speed region.

### 3.3. Wear Rate

**Figure 11:**
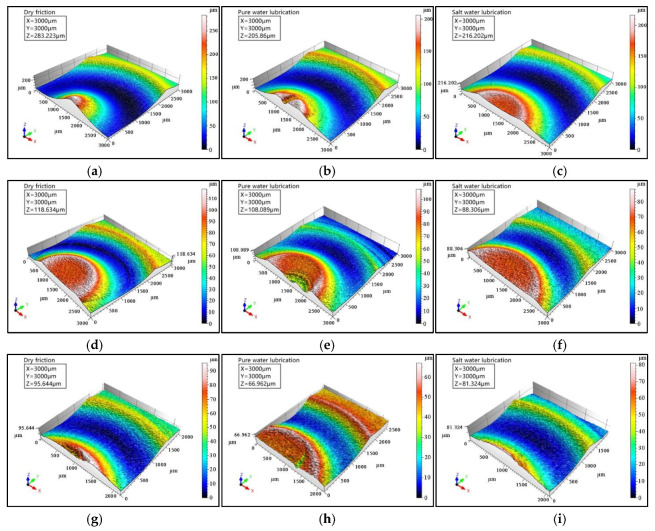
Three-dimensional surface morphologies of UHMWPE under dry friction, water-unsaturated lubrication and saltwater-unsaturated lubrication conditions: (**a**–**c**) 110 N load and 500 r/min speed, (**d**–**f**) 50 N load and 1100 r/min speed, and (**g**–**i**) 50 N load and 500 r/min speed.

The wear rate is considered to be a key indicator for studying the wear performance of the rubbing pair. In this experiment, the wear volume and wear rate were derived by marking the width of the wear scar using the ST400 surface profiler produced by NANOVEA of the United States, which was a 3D non-contact high-precision surface topography measuring instrument that could achieve a resolution of less than 2 nm. Figure 11 shows 3D surface morphologies of UHMWPE in the three-friction conditions. Taking the 50 N load and 500 r/min speed as the benchmark, it can be seen that under water- and brine-unsaturated lubrication, the increase in the peak-to-valley height difference when the load is increased to 110 N is far greater than that when the speed is increased to 1100 r/min. This indicated that for unsaturated lubrication, the effect of load on the wear rate of UHMWPE was more significant than that of speed.

Under 110 N load and 500 r/min speed, the height difference of UHMWPE under dry friction was the largest, exceeding 280 μm, followed by that under saltwater-unsaturated lubrication, about 220 μm, and that under pure water-unsaturated lubrication was the smallest, which was 210 μm. When the case changed to 50 N load and 1100 r/min speed, the height difference of UHMWPE under dry sliding was still the largest, but that under brine-unsaturated lubrication was smaller than that under pure water-unsaturated lubrication. This is different from the general belief that the wear resistance of UHMWPE under pure water lubrication is better than that under saltwater lubrication, indicating that the wear mechanisms of UHMWPE under unsaturated lubrication and saturated lubrication are different.

**Figure 12:**
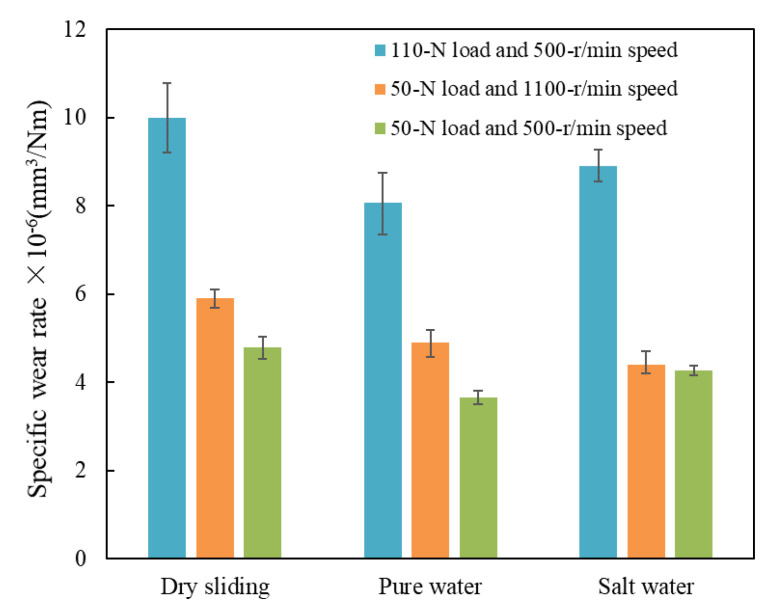
Specific wear rates of UHMWPE under dry sliding, water lubrication and saltwater lubrication at different sliding variables.

The formula for the fraying volume *V* is as follows:

(2) $$V=B\left[R^{3}\arcsin\frac{b}{2R}-\frac{b}{2}\sqrt{R^{2}-\frac{b^{2}}{4}}\right],$$

where *B* is the width of the grinding mark (mm), *R* friction pair ring radius (mm), and *b* the sample width (mm). The specific wear rate *K* (mm^3^/(N·m)) is:

(3) $$K=\frac{V}{L\cdot d},$$

where *d* is the sliding distance (m) and *L* the applied load (N). According to the calculation results, the bar graphs of *K* under dry sliding, water-unsaturated lubrication and brine-unsaturated lubrication were drawn, as shown in Figure 12.

It can be seen in Figure 12 that for the same sliding variables, as it should be, the dry sliding wear rate is the largest. For the sliding variables of 50 N load and 1100 r/min speed, the wear rate of UHMWPE under brine-unsaturated lubrication was the smallest, which was 4.4 × 10^−6^ mm^3^/(N·m) and which was 10% lower than that of water-unsaturated lubrication. While under the sliding variables of 110 N load and 500 r/min speed, the water-unsaturated lubrication had the smallest wear rate, which was 9.32% lower than that of brine-unsaturated lubrication.

Under unsaturated lubrication, the wear rate in brine was lower than that in water at high speed, which was exactly the opposite of the saturated lubrication [11]. It was mainly due to the fact that water-unsaturated lubrication was close to dry sliding at high speed, while salt granules precipitated from the saltwater during brine-unsaturated lubrication played a ball effect. However, at high load conditions, the salt granules were squeezed into the surface of the sample, and scratches were produced under unsaturated lubrication, resulting in a higher wear rate than that under water-unsaturated lubrication.

## 4. Conclusions

The tribological behaviors of UHMWPE under unsaturated lubrication of water and brine were studied, and the following conclusions can be drawn from the present study:

1. Under certain liquid drop (about 150–170 mL/h) lubrication, the lubrication form of UHMWPE gradually transited from saturated lubrication to unsaturated lubrication with the increase in applied load or sliding speed due to the evaporation of water caused by frictional heat. When the load exceeds 70 N or the speed exceeds 700 r/min, UHMWPE is in unsaturated lubrication, and its tribological behaviors are better than those of dry sliding. Compared with those of dry sliding, the friction coefficients of UHMWPE under water-unsaturated lubrication are reduced by 50.07–69.43% and 51.81–63.92% at different loads and speeds, respectively, while those under brine-unsaturated lubrication are reduced by 42.34–56.43% and 39.00–68.45%, respectively. The wear rate of UHMWPE under water-unsaturated lubrication is 17.11–23.64% lower than that under dry sliding, and the wear rate of UHMWPE under saltwater-unsaturated lubrication is 10–25.42% lower than that under dry sliding.
2. The anti-friction property in brine-unsaturated lubrication is better than that in water-unsaturated lubrication due to the rolling effect of precipitated salt granules at high speed. When the sliding speed is 1100 r/min, the friction coefficient of UHMWPE under brine-unsaturated lubrication is 32.44% lower than that under water-unsaturated lubrication.
3. The wear resistance of UHMWPE under brine-unsaturated lubrication is better than that in water-unsaturated lubrication at high speed, and the wear rate of UHMWPE under brine-unsaturated lubrication was 10% lower than that under water-unsaturated lubrication at 1100 r/min speed. However, at high load conditions, UHMWPE has better wear resistance under water-unsaturated lubrication.

## Figures and Tables

**Table 1:** Basic technical performances of ultra-high molecular weight polyethylene.

Molecular Weight	Density (g/cm)	Distortion Temperature (°C)	Brittle Temperature (°C)	Tensile Modulus (MPa)	Elongation at Break (%)	Impact Strength (kJ·m^2^)	Thermal Conductivity (w/(m·k))
4.5 × 10^6^	0.93	85	−70	34	350	130	0.41

**Table 2:** The main ionic components of the brine.

Ions	Concentration (g/L)
Na^+^	0.055
Cl^−^	0.430
SO_4_^2−^	1.524
Ca^2+^	0.689
Mg^2+^	0.141
CO_3_^2−^	0.096
NO_3_^−^	0.042

**Table 3:** Temperatures of UHMWPE surface at 500 r/min speed under different friction conditions.

Loads (N)	Temperature (°C) under Different Friction Conditions		
	Dry Friction	Water Lubrication	Brine Lubrication
30	43.42 ± 4.21	30.64 ± 3.20	31.11 ± 3.53
50	49.75 ± 3.09	33.23 ± 3.51	34.94 ± 3.70
70	57.62 ± 5.20	37.14 ± 2.88	37.16 ± 3.06
90	61.21 ± 4.55	41.57 ± 3.64	42.81 ± 3.40
110	65.17 ± 3.98	44.36 ± 2.31	44.28 ± 4.33

**Table 4:** Temperatures of UHMWPE surface at 50 N load under different friction conditions.

Speed (r/min)	Temperature (°C) under Different Friction Conditions		
	Dry Friction	Water Lubrication	Brine Lubrication
300	38.15 ± 3.25	25.17 ± 2.93	27.26 ± 2.60
500	49.75 ± 3.09	33.23 ± 3.51	35.94 ± 3.70
700	61.26 ± 4.98	45.36 ± 3.59	46.21 ± 3.66
900	76.74 ± 5.21	60.23 ± 2.07	59.95 ± 6.13
1100	88.38 ± 5.55	77.72 ± 3.68	78.61 ± 2.33

## Data Availability

Data are contained within the article.
